# 4D-STEM Ptychography for Electron-Beam-Sensitive Materials

**DOI:** 10.1021/acscentsci.2c01137

**Published:** 2022-11-21

**Authors:** Guanxing Li, Hui Zhang, Yu Han

**Affiliations:** Advanced Membranes and Porous Materials Center, Physical Science and Engineering Division, King Abdullah University of Science and Technology, Thuwal 23955-6900, Saudi Arabia

## Abstract

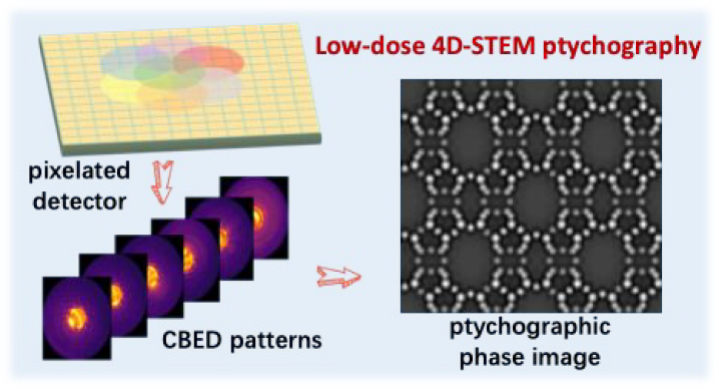

Recent advances in high-speed pixelated electron detectors have substantially facilitated the implementation of four-dimensional scanning transmission electron microscopy (4D-STEM). A critical application of 4D-STEM is electron ptychography, which reveals the atomic structure of a specimen by reconstructing its transmission function from redundant convergent-beam electron diffraction patterns. Although 4D-STEM ptychography offers many advantages over conventional imaging modes, this emerging technique has not been fully applied to materials highly sensitive to electron beams. In this Outlook, we introduce the fundamentals of 4D-STEM ptychography, focusing on data collection and processing methods, and present the current applications of 4D-STEM ptychography in various materials. Next, we discuss the potential advantages of imaging electron-beam-sensitive materials using 4D-STEM ptychography and explore its feasibility by performing simulations and experiments on a zeolite material. The preliminary results demonstrate that, at the low electron dose required to preserve the zeolite structure, 4D-STEM ptychography can reliably provide higher resolution and greater tolerance to the specimen thickness and probe defocus as compared to existing imaging techniques. In the final section, we discuss the challenges and possible strategies to further reduce the electron dose for 4D-STEM ptychography. If successful, it will be a game-changer for imaging extremely sensitive materials, such as metal–organic frameworks, hybrid halide perovskites, and supramolecular crystals.

## Abstract 2

Electron ptychography has regained widespread research attention due to the advent of 4D-STEM, showing potential advantages for atomic-resolution imaging of electron-beam-sensitive materials.

## 1 Introduction

Transmission electron microscopy (TEM) integrates diffraction, imaging, and spectroscopy and plays a vital role in material chemistry.^1−6^ Modern electron microscopes equipped with spherical aberration correctors can probe the crystallographic, physical, and chemical properties of materials with a subangstrom spatial resolution. However, many materials are sensitive to electron beam irradiation, and their high-resolution TEM imaging has long been challenging due to electron-beam-induced structural damage.^7−9^ Typical electron-beam-sensitive materials (or “beam-sensitive materials”) include materials with porous structures, organic components, or weak chemical bonds, such as metal–organic frameworks (MOFs), covalent–organic frameworks, organic–inorganic hybrid perovskites, and supramolecular crystals. These materials can only withstand dozens of electrons per square angstrom (e^–^/Å^2^); thus, imaging their intrinsic structures requires ultralow electron doses (i.e., below the thresholds) to avoid structural damage. Conventional TEM cannot produce useful images at such low electron doses.

Over the past several years, the advent of high-efficiency cameras and detectors combined with new imaging strategies has enabled electron microscopy imaging of beam-sensitive materials in both TEM and scanning TEM (STEM) modes. In the TEM mode, high-resolution TEM (HRTEM) with ultralow electron doses has been achieved by using direct-detection electron-counting cameras.^10−13^ The development of a suite of image acquisition and image processing methods has significantly improved the efficiency of ultra-low-dose HRTEM, making it an almost routine method.^11^ This method can produce images at electron doses as low as a few e^–^/Å^2^, which is particularly useful for imaging materials extremely sensitive to the electron beam, such as MOFs and hybrid perovskites.^12,14^ The disadvantage of HRTEM is that the image contrast cannot be directly interpreted, whereas image processing to make it interpretable requires expertise and often results in artifacts. In the STEM mode, integrated differential phase contrast STEM (iDPC-STEM) has emerged as an efficient low-dose technique for imaging beam-sensitive materials, including zeolites, covalent–organic frameworks, and MOFs.^12,15−19^ Compared with HRTEM, the image contrast of iDPC-STEM is easier to interpret. On the other hand, however, iDPC-STEM requires precise focusing of the electron beam to achieve atomic resolution, which could result in a lower success rate because structural damage is likely to occur during the fine-tuning process of the beam focus, especially when the specimen is highly beam-sensitive. In addition, the image quality of iDPC-STEM degrades rapidly as the specimen thickness increases.

A technique combining the advantages of ultra-low-dose HRTEM and iDPC-STEM while avoiding their disadvantages would be ideal for imaging beam-sensitive materials. Specifically, it should produce easily interpretable images at low electron doses and be highly tolerant to focusing conditions and specimen thickness. In this context, we propose that electron ptychography based on the four-dimensional (4D)-STEM dataset may be a candidate for this purpose, although there are still some practical obstacles to overcome.

The concept of ptychography was initially invented in 1969 to provide a solution to the crystallographic phase problem and was later developed into a general coherent imaging method applicable to various irradiation sources, including visible light, X-rays, extreme ultraviolet, and electrons.^20−22^ In theory, ptychography is unaffected by lens aberrations or limited numerical apertures, enabling “super resolution” relative to conventional lens imaging. However, limited by the hardware development level and computing ability, electron ptychography has not demonstrated its anticipated ability for a long time. It is only in recent years that electron ptychography has regained widespread research interest due to a dramatically improved computing ability and the invention of high-performance electron detectors. Remarkably, electron ptychography has provided the highest-resolution images ever obtained, reaching the resolution limit set by the lattice vibrations of the specimen.^23^

Although atomic-resolution (S)TEM imaging has become routine in the past decade, making the resolution no longer the most crucial consideration, electron ptychography still offers several other advantages particularly helpful for imaging beam-sensitive materials. First, electron ptychography reconstructs the object transmission function from diffraction patterns, enabling the generation of directly interpretable phase images. Compared with images formed through other mechanisms, the phase image has stronger contrast, thus requiring a lower electron dose and having the ability to probe heavy and light elements simultaneously. Second, electron ptychography can be performed at varying optical configurations, allowing the use of a defocused beam to generate diffraction patterns. Therefore, the discussed dose-constrained focusing problem of iDPC-STEM can be circumvented with electron ptychography to improve the imaging efficiency for beam-sensitive materials. Third, unlike conventional (S)TEM imaging methods that require the specimen to be very thin to avoid multiple scattering, electron ptychography can minimize multiple scattering effects by employing the multislice method in the reconstruction algorithm and has a greater tolerance for the specimen thickness.

In TEM, electron ptychography can be performed based on a 4D-STEM dataset, generated by scanning a convergent electron beam across the specimen in a two-dimensional (2D) raster fashion while recording the 2D diffraction data produced at each scan position with a pixelated detector. The unique optical geometry of STEM, combined with the rapid development of fast detectors, has revitalized the application of ptychography in electron microscopy. In this Outlook, we briefly describe the standard procedures for 4D-STEM data acquisition and processing and introduce some application examples of 4D-STEM ptychography. Next, we demonstrate that 4D-STEM ptychography can be implemented at low-dose conditions for beam-sensitive materials, using mordenite (MOR), an aluminosilicate zeolite, as a model material. Finally, we discuss the potential, feasibility, and requirements of applying 4D-STEM ptychography for imaging materials with extremely high beam sensitivity, such as MOFs. Should one be interested in more technical details of 4D-STEM, we recommend two recent review articles.^24,25^

## 2 Data Acquisition and Processing of 4D-STEM Ptychography

**Figure 1:**
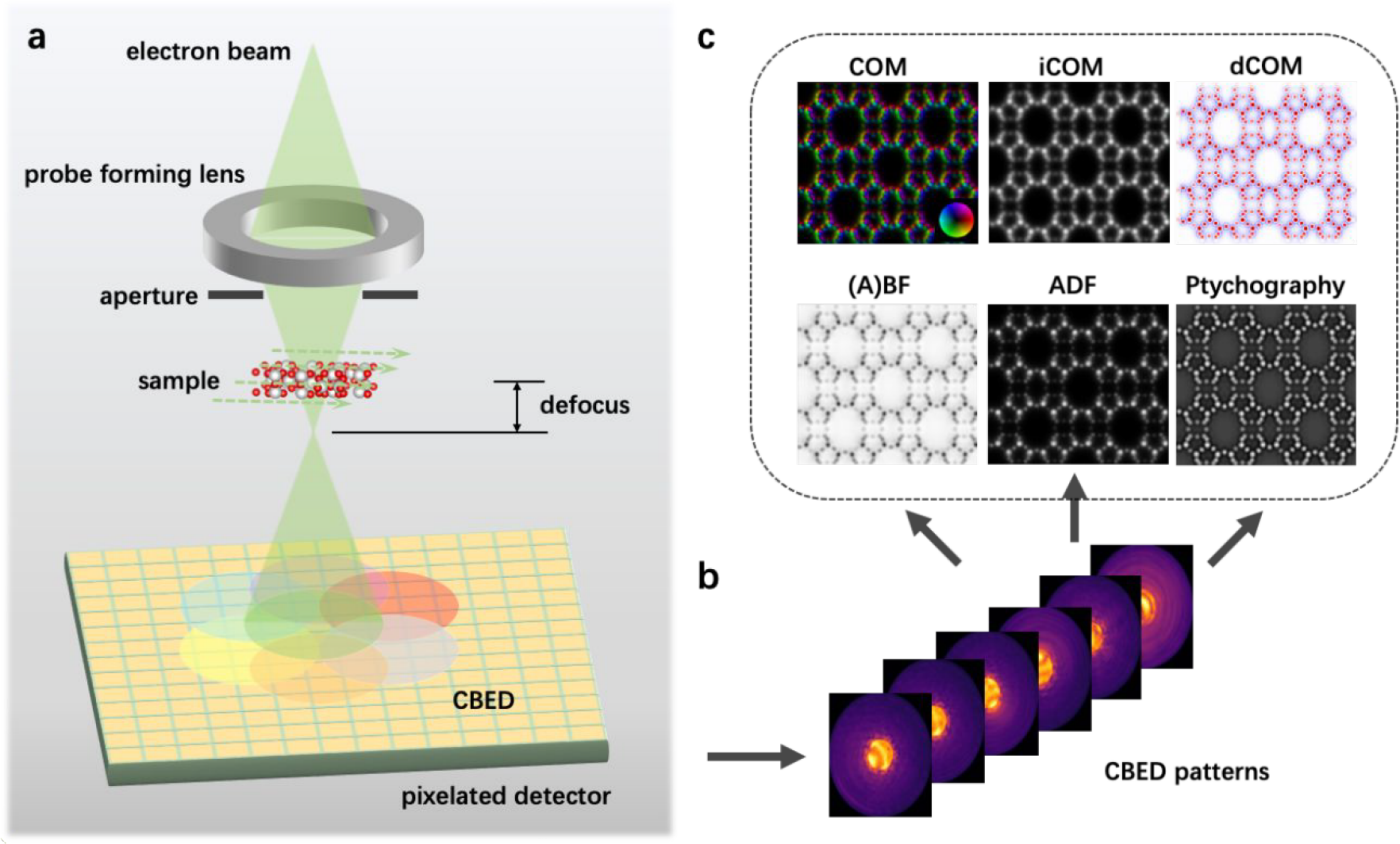
Schematic illustration of 4D-STEM. (a) A typical electron optical configuration for 4D-STEM. (b) 4D-STEM dataset consisting of a series of convergent-beam electron diffraction (CBED) patterns. (c) Various images computed from the 4D-STEM dataset using different signals or imaging theories.

A 4D-STEM dataset is a series of convergent-beam electron diffraction (CBED) patterns collected during the scanning of a convergent electron beam (probe) over a specimen (Figure 1a,b). The 4D-STEM ptychography employs interferences between diffraction disks in the CBED patterns for phase reconstruction. Therefore, a suitable convergent semiangle (α) should be selected to realize the overlap of diffraction disks. Moreover, successful ptychography reconstructions typically require high data redundancy, preferably greater than 60%.^26^ To achieve redundancy, the probe and scanning-step sizes should be chosen appropriately to ensure that two adjacent scan regions have a sufficient overlap. Compared with a focused probe, defocused probes allow larger scanning-step sizes to cover an area with lower electron doses or view a larger field with the same amount of data. The ability to achieve atomic-resolution reconstructions using defocused probes is the most significant advantage of 4D-STEM ptychography for imaging beam-sensitive materials. The camera length must also be properly set to balance the pixel size in reciprocal space and the range of recorded scattering angles. Too-small camera lengths result in poor sampling within the CBED patterns, whereas too-large camera lengths limit the achievable reconstruction resolution, both adversely affecting the quality of the reconstructed phase images.

In addition to the proper choice of imaging conditions, high-performance electron detectors are critical for successful 4D-STEM ptychography reconstruction, especially when only a limited electron dose can be applied (e.g., when the specimen is beam-sensitive). Direct electron detectors with a high detection quantum efficiency are typically required to obtain high-quality 4D-STEM data with a good signal-to-noise ratio (SNR). The highest available frame rate of the detector is another critical factor. In most instances, a higher frame rate (faster detector) is desirable because it takes less time to complete the scan, reducing the sample drift that can severely affect reconstruction. According to recent studies, frame rates greater than 1000 frames per second (fps) are typically required to achieve good reconstructions.^23,27^ In addition, the detector should have a wide dynamic range to record high-intensity bright-field (BF) signals and low-intensity high-angle signals simultaneously. The ideal electron detector for 4D-STEM should combine a high dynamic range (usually corresponding to large pixel sizes), large pixel numbers, and fast data readout speed. The currently available detectors exhibit trade-offs between these parameters and should be carefully selected based on the specific application requirements. A recent review detailed the introduction and comparison of various 4D-STEM electron detectors.^28^

A 4D-STEM dataset contains complete diffraction information and can be processed using different virtual detector configurations and algorithms to reconstruct various types of images (Figure 1c). For example, virtual annular detectors with different collection angles can be defined to calculate BF, annular BF (ABF), and annular dark-field (ADF) images from the 4D-STEM dataset. Likewise, the center of mass (COM) and its extensions, the integrated COM (iCOM) and the differentiated COM (dCOM), corresponding to the projected electrical field, electrostatic potential, and charge density of the specimen, respectively, can be calculated using the 4D-STEM dataset. The calculated COM-based images are similar to the differential phase contrast-based (i.e., DPC, iDPC, and dDPC) images acquired using segmented detectors but have higher accuracy.^29^ In addition to rendering images based on conventional mechanisms, the 4D-STEM dataset can be used to calculate ptychographic phase images using various algorithms. From the same dataset, ptychography usually produces the clearest images compared with images from other imaging theories.^30^ The following paragraphs briefly introduce some representative ptychography algorithms.

An early computational method for retrieving phase information in ptychography is the Wigner distribution deconvolution method (WDDM)^31,32^ proposed by Rodenburg et al. This method has demonstrated success in optical and X-ray experiments.^33,34^ For the reconstruction of thin specimens that meet weak phase object approximation, the WDDM can be simplified to the single sideband (SSB) method. However, the WDDM is prone to error or failure when the noise level of the data is high, although some efforts have been made to address this problem.^35^

The WDDM was later replaced by a new iterative algorithm, the ptychographical iterative engine (PIE). The PIE algorithm uses a known probe function to calculate the target sample object function^36^ and has been successfully applied in optical experiments,^37^ X-ray experiments,^38^ and electron microscopy.^39^ Because the probe function cannot be accurately obtained in most cases, extra efforts are required to retrieve the probe function.^40,41^ Later, an extended PIE, called “ePIE,”^42^ was developed, which does not rely on a known probe function. In the reconstruction process using ePIE, the object and probe functions are both updated until the iteration is convergent. Other algorithms, such as pcPIE^43^ and 3PIE,^44^ were derived from ePIE.

When the noise level of the data is high, the maximum likelihood (ML) method^45^ exhibits robust reconstruction. The ML method updates the exit wave by maximizing the possibility of observing the intensity distribution in the experimental diffraction patterns under specific noise models, making it outperform other algorithms, including ePIE and the difference map method.^46^ In addition to these widely used algorithms, several other reconstruction algorithms have been developed, such as regularized optimization for ptychography^47^ and global ptychographic iterative linear retrieval using Fourier transforms.^48^

The redundancy of the 4D-STEM dataset allows the correction of experimental imperfections and aberrations through reconstruction. For example, position correction^43,49^ and zone-axis correction^50^ have been realized using modified algorithms. In addition, the multislice^44,51^ and mixed-state methods,^52,53^ which can be integrated into iterative algorithms (e.g., ePIE and ML algorithms), have been developed to address the problems of multiple scattering associated with thick specimens and the partial incoherence of electron beams, respectively. The multislice method divides the samples into several thin slices during the reconstruction process, and the structures of these slices can be separately retrieved. Thus, when used for thick specimens, the multislice method not only improves the lateral resolution but also provides resolving power along the projection direction.

## 3 Applications of 4D-STEM Ptychography

**Figure 2:**
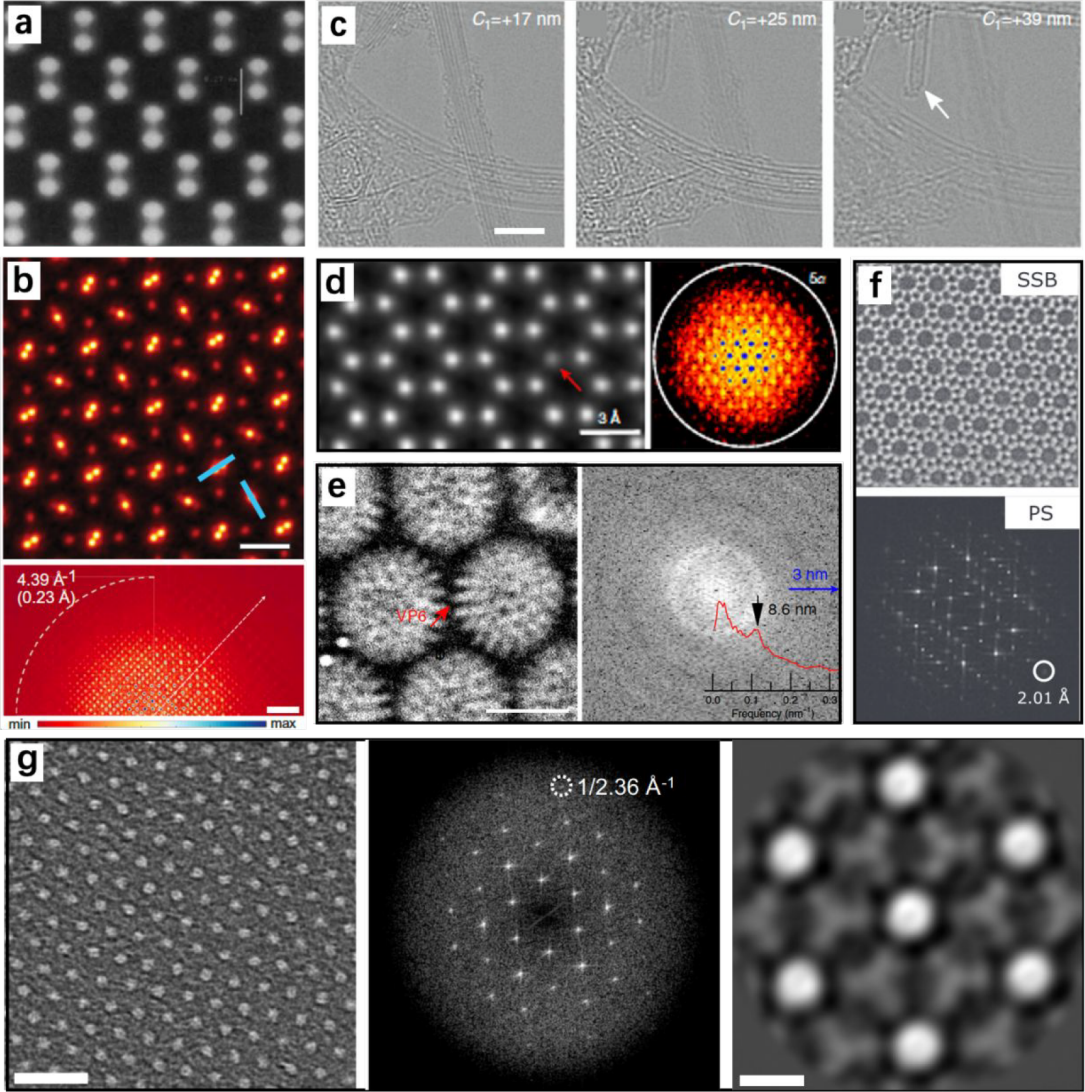
Representative application examples of 4D-STEM ptychography. (a) Pioneering work of electron ptychography to resolve Si columns 0.136 nm apart. Scale bar, 0.27 nm. Reproduced with permission from ref (55). Copyright 1995 Springer Nature. (b) Phase image of [001]-oriented PrScO_3_ reconstructed using the multislice method (upper) and its Fourier transform (lower). Scale bars, 2 Å and 1 Å^–1^, respectively. Reproduced with permission from ref (23). Copyright 2021 American Association for the Advancement of Science. (c) Optical sectioning images of carbon nanotubes at different defocus values using the Wigner distribution deconvolution method. The arrow indicates a small carbon nanotube that becomes visible at a defocus of +39 nm. Scale bar, 5 nm. Reproduced with permission from ref (57). Copyright 2016 Springer Nature. (d) Phase image of the monolayer MoS_2_ (left) and corresponding diffractogram (right), demonstrating an information limit close to 5α. Reproduced with permission from ref (27). Copyright 2018 Springer Nature. (e) Cryoptychographic phase image of rotavirus double-layered particles (left) and its power spectrum with the radial average as the inset (right). Scale bar, 50 nm. Reproduced with permission from ref (59). Copyright 2020 Springer Nature. (f) Phase image of zeolite ZSM-5 reconstructed using single sideband (SSB)-based ptychography (upper) and the corresponding power spectrum (lower). Reproduced with permission from ref (60). Copyright 2020 American Institute of Physics. (g) SSB-based ptychography phase image of a Hf-based metal–organic layer material (left), its Fourier transform (middle), and the processed image by averaging 70 unit cells (right). Scale bars, 5 nm (left) and 1 nm (right). Reproduced with permission from ref (72). Copyright 2022 Springer Nature.

In the pioneering studies^54,55^ on STEM ptychography, Nellist, Rodenburg, and co-workers resolved Si columns 0.136 nm apart, using a microscope with a point resolution of only 0.42 nm (Figure 2a). In another study, they collected diffraction data from gold nanoparticles using a 30 kV scanning electron microscope with a standard resolution of 1.2 nm. The ptychography reconstruction using ePIE successfully resolved Au lattice fringes with a *d*-spacing of 0.236 nm, corresponding to a 5-fold increase in resolution.^56^ This work demonstrated the feasibility of high-resolution imaging using a low-energy electron beam under suboptimal conditions, implying the great potential of ptychography for imaging beam-sensitive materials. Recently, Chen et al. reported a record high resolution of ∼0.2 Å obtained on a 21 nm thick PrScO_3_ specimen using ML-based electron ptychography (Figure 2b).^23^

Ptychography can provide structural information along the beam propagation direction, which is a significant advantage compared with conventional (S)TEM modes that primarily produce projection images. For example, Yang et al. used the WDDM algorithm to reconstruct the phase images of a specimen containing carbon nanotubes (CNTs). The reconstruction showed the distribution of CNTs at different depths, revealing the presence of a small CNT that could hardly be observed via other imaging methods (Figure 2c).^57^ In Chen et al.’s work mentioned above, using a simulated dataset, they demonstrated that a depth resolution of 0.9 nm could be achieved to locate the position of an interstitial dopant atom in a PrScO_3_ lattice.^23^

## 4 Exploration of 4D-STEM Ptychography for Beam-Sensitive Materials

Although 4D-STEM ptychography is a powerful low-dose imaging technique, its application for beam-sensitive materials remains largely unexplored. The difficulties primarily lie in the following problems. First, acquiring data from beam-sensitive materials without destroying their inherent structure is nontrivial, requiring a carefully designed suite of methods to minimize the consumed electron dose.^11^ Second, the limited availability of high-performance electron detectors required for 4D-STEM ptychography is another obstacle to its more comprehensive application. Third, the low electron dose required for beam-sensitive materials results in poor SNR of the obtained 4D-STEM data, whereas most algorithms, especially iterative ones, require high SNR data to achieve reliable convergent results.

The initial attempts to image sensitive materials using 4D-STEM ptychography focused on 2D and biological materials. In these attempts, in addition to a low electron dose, low accelerating voltages or cryogenic temperatures were often used to reduce the structural damage caused by knock-on or heating effects. For example, Pennycook et al. collected 4D-STEM data for bilayer graphene at 60 kV to demonstrate that the contrast of the ptychography phase images was superior to that of the high-angle ADF images.^30^ The same conclusion was obtained in a study using 80 kV 4D-STEM to image 2D-transition metal dichalcogenide MoS_2_.^58^

In another study, Jiang et al. performed ptychography for monolayer MoS_2_ using 4D-STEM data collected at 80 kV and achieved a super resolution of 0.39 Å, corresponding to five times the convergence semiangle (Figure 2d).^27^ The electron doses in these studies were on the order of several thousand e^–^/Å^2^. In fact, as 2D materials primarily follow the knock-on damage mechanism, higher electron doses can be used as long as the electron beam energy (i.e., the accelerating voltage applied) is sufficiently low. For example, Chen et al. revealed a 4% lattice mismatch in a bilayer WS_2_/MoSe_2_ heterostructure using ptychography involving the mixed-state method,^53^ where the 4D-STEM data were acquired at 80 kV with a total electron dose exceeding 15 000 e^–^/Å^2^.

Zhou et al. reported the imaging of biological specimens (rotavirus particles) by combining 4D-STEM ptychography with cryoelectron microscopy.^59^ The results revealed that the contrast transfer was significantly improved in the low spatial frequency range (Figure 2e), providing more morphological information than conventional HRTEM. Moreover, the total electron dose was successfully reduced to 22.8 e^–^/Å^2^ due to a greatly defocused beam enabled by ptychography.

Compared with 2D materials and biological specimens, 3D crystalline beam-sensitive materials, such as zeolites, MOFs, and halide perovskites, are much more challenging to image with (S)TEM for two reasons. First, these materials generally follow a radiolysis damage mechanism, exhibiting minimal electron dose tolerances regardless of the electron beam energy. Therefore, adjusting the accelerating voltage cannot improve their stability under electron irradiation. Second, unlike 2D crystals or biological specimens that can often be imaged directly without orientation adjustment, 3D crystals require an additional “zone axis alignment” step prior to imaging. For beam-sensitive materials, their structures can be easily damaged during this step if the alignment cannot be accomplished quickly enough. Because of these practical obstacles, 4D-STEM ptychography has rarely been used to study 3D crystalline beam-sensitive materials.

O’Leary et al. performed 4D-STEM ptychography reconstruction of zeolite ZSM-5 using the SSB algorithm.^60^ They demonstrated that reconstruction could be successful even with maximal binning of the 4D-STEM data, providing a promising strategy for reducing beam damage. With this strategy, they were able to acquire data with a low electron dose of ∼1000 e^–^/Å^2^ and achieve ∼2 Å resolution in the reconstructed image (Figure 2f). However, such a resolution is only comparable to that of conventional (S)TEM imaging, and it is insufficient to resolve oxygen in the zeolite framework or related local structural features. Given that the electron dose chosen in this study was not optimized, we expect higher resolutions to be achieved for zeolite materials by identifying optimal data acquisition methods and reconstruction algorithms. We believe it is time to explore the potential of 4D-STEM ptychography for beam-sensitive materials, as the recently developed one-step automatic method removes the obstacle of “zone axis alignment”.^11^

To demonstrate the potential advantages of 4D-STEM ptychography for imaging beam-sensitive materials, we performed imaging simulations using an inorganic porous material, zeolite MOR, as the model material. The abTEM code in the Python library was used to simulate 4D-STEM datasets.^61^ The accelerating voltage and convergence semiangle of the electron beam were set to 300 kV and 15 mrad, respectively. The scanning-step size in real space was set to 0.25 Å and the pixel size in reciprocal space to 0.01 Å^–1^ × 0.01 Å^–1^, and 20 frozen configurations were used to account for thermal diffuse scattering. The simulated datasets were used to reconstruct various types of images using the py4DSTEM^62^ or self-developed code.

Through the simulation, we compared 4D-STEM ptychography with the state-of-the-art STEM imaging technique for zeolite materials (i.e., iDPC-STEM) in terms of robustness to changes in specimen thickness, electron dose, and defocus value. Given the same image formation mechanism,^29^ iDPC-STEM images can be represented by iCOM images reconstructed from the simulated datasets. Two types of ptychography images are generated using SSB and ML algorithms, respectively.

**Figure 3:**
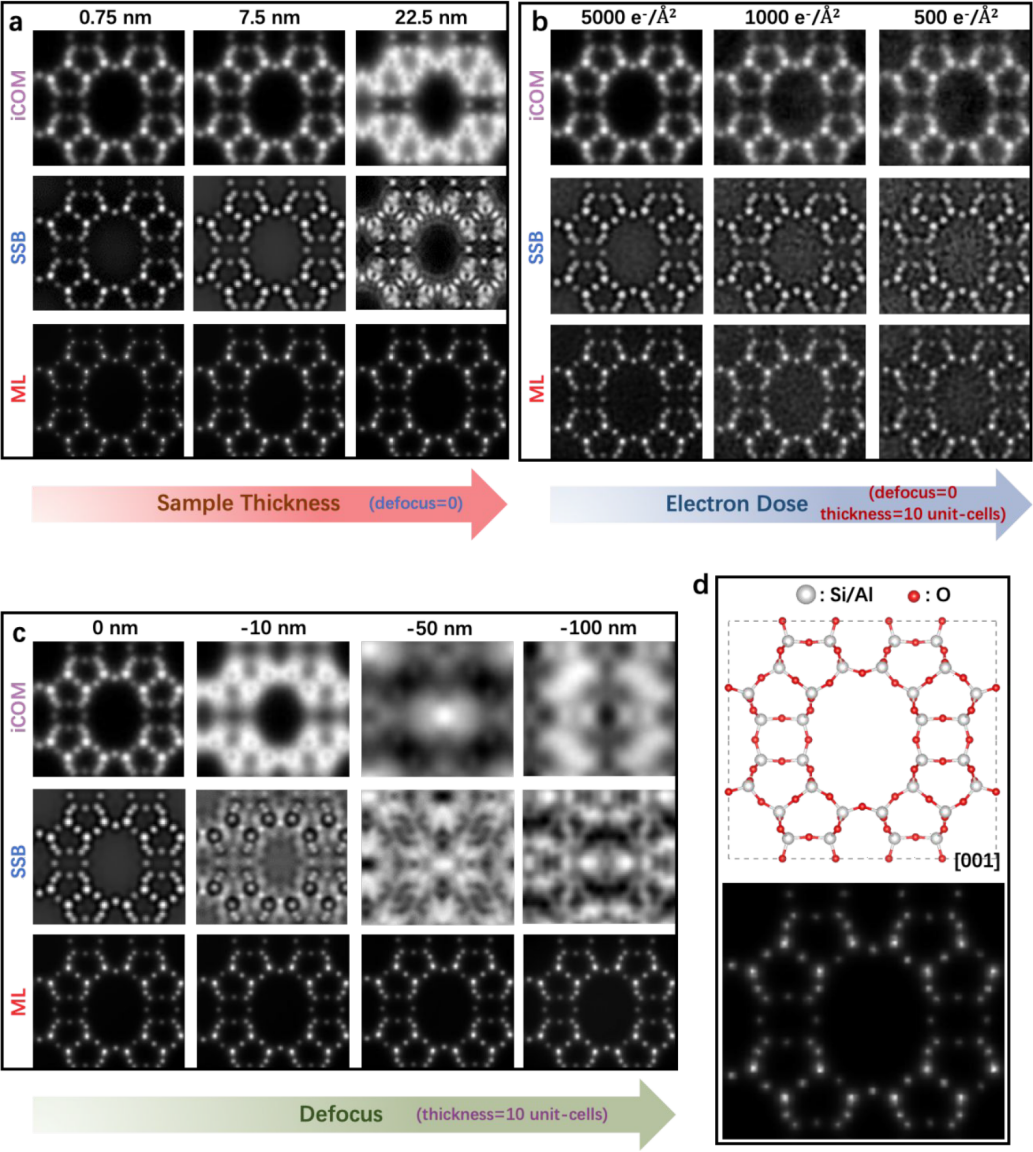
Image reconstruction from a simulated 4D-STEM dataset of zeolite mordenite (MOR) along the [001] zone axis. Investigating the effect of the (a) specimen thickness, (b) electron dose, and (c) defocus value on iCOM, single sideband-ptychography, and maximum likelihood-ptychography employing the multislice method. (d) [001]-projected structural model of zeolite MOR (upper) and corresponding calculated electrostatic potential map (lower).

To investigate how specimen thickness affects reconstruction, we constructed three models of the [001]-projected MOR structure that are 0.75, 7.5, and 22.5 nm thick, corresponding to 1, 10, and 30 unit cells, respectively. The results reveal that, for thin specimens (0.75 and 7.5 nm), the iCOM images match the projected structural model and simulated electrostatic potential map of MOR, displaying individual Si and O atomic columns at high resolution (Figure 3a,d). However, the iCOM image quality severely deteriorates with increased specimen thickness. For the 22.5 nm thick specimen, the iCOM image cannot resolve all framework atoms and is no longer directly interpretable (i.e., it does not match the structural model; Figure 3a). Likewise, the ptychography images reconstructed using the SSB method exhibit high quality only when the specimen is extremely thin (Figure 3a). This is understandable because the SSB algorithm also relies on the weak phase object approximation. Remarkably, the ML algorithm involving the multislice method is very robust, displaying substantial tolerance for the specimen thickness variation. The images reconstructed for various specimen thicknesses exhibit similarly high quality, with all framework atoms, including oxygen atoms, clearly resolved (Figure 3a). These simulation results demonstrate that, unlike iDPC-STEM, which requires a very thin specimen to obtain directly interpretable atomic-resolution images, 4D-STEM ptychography can be used for thick samples with proper reconstruction methods.

We also investigated the effect of the electron dose on reconstruction through simulations. Specifically, we fixed the specimen thickness at 7.5 nm (i.e., 10 unit cells) while adding various degrees of randomly distributed Poisson noise to the 4D-STEM dataset to simulate various electron doses.^61^ At a relatively high dose of 5000 e^–^/Å^2^, iCOM, SSB, and ML provide good results, resolving the structural details. At lower electron doses (i.e., 1000 and 500 e^–^/Å^2^), all three methods can still produce phase images to resolve the framework atomic columns, albeit with higher noise levels, especially for the iCOM images (Figure 3b). The excellent performance of iCOM and ptychography at low electron doses can be attributed to their high electron utilization efficiencies. By comparison, ptychography is even more robust than iCOM (or iDPC).

The most important potential advantage of ptychography for imaging beam-sensitive materials is that it can work with a defocused electron beam. To verify this advantage, we performed simulations using a fixed specimen thickness of 7.5 nm while varying the defocus value of the electron probe from 0 to −100 nm (Figure 3c). The results indicate that iCOM and SSB-based ptychography are sensitive to the defocus condition. The image quality is greatly reduced when using a slightly defocused probe (−10 nm). At relatively larger defocus values of −50 and −100 nm, the reconstructed images display strange contrasts that deviate from the MOR structure and cannot be directly interpreted. Notably, ML-based ptychography exhibits high tolerance to defocus variations. Nearly identical reconstruction results were obtained when the defocus was tuned in the range from 0 to −100 nm (Figure 3c). These results demonstrate that a well-chosen ptychography method can largely address the dose-constrained focusing problem associated with extremely beam-sensitive materials by using an unfocused electron beam for data acquisition.

Encouraged by the simulation results, we conducted a preliminary experimental attempt to explore the practical effects of 4D-STEM ptychography for imaging zeolite structures at low electron doses. The 4D-STEM experiment was performed on a 300 kV aberration-corrected TEM instrument (FEI Titan Cubed Themis Z) equipped with an EMPAD detector (128 × 128 pixels and 1000 fps), using a zeolite material as the specimen. The total electron dose in the experiment was 1500–3000 e^–^/Å^2^, which is lower than that used in conventional STEM imaging of zeolite materials.^63^

Using the acquired 4D-STEM dataset, we reconstructed ADF, ABF, iCOM, and ML-based ptychography images. As ADF and ABF have relatively low electron utilization efficiency leading to poor SNR at the low-dose conditions used in this experiment, the reconstructed ADF and ABF images show SNR-limited resolution. The reconstructed iCOM image has a higher resolution than ADF and ABF images due to the improved SNR, which is consistent with observations from a series of studies on low-dose STEM imaging.^64−66^ At the resolution of the iCOM image, some (but not all) Si/Al atoms on the zeolite framework can be resolved, where the limited resolution can be attributed to the insufficient thinness of the prepared zeolite specimens. Significantly, ptychography provides much better results compared with the other imaging modes. The ptychographic phase images resolve all framework atoms, including oxygen, at a subangstrom resolution, which perfectly agrees with the simulation. We will report these new findings along with the experimental details in a separate research article in the future.

## 5 Conclusions and Outlook

The imaging simulations demonstrate that 4D-STEM ptychography has several advantages over conventional (S)TEM, including greater tolerance to the specimen thickness and defocus values and better performance at low doses. The preliminary experimental results on zeolite structures confirm that 4D-STEM ptychography can achieve a subangstrom resolution at the electron dose level of 1500–3000 e^–^/Å^2^.

Zeolites are generally considered beam-sensitive (but not very sensitive) and can typically withstand a few thousand electrons per square angstrom under 300 kV STEM conditions, depending on the specific structure and Si/Al ratio. Although dose levels of a few thousand electrons per square angstrom are quite low in conventional atomic-resolution (S)TEM, they are too high for extremely electron-beam-sensitive materials, such as MOFs and hybrid perovskites. Previous iDPC-STEM experiments of MOFs have used electron doses in the range 40–200 e^–^/Å^2^.^12,67−69^ Note that the dose thresholds for MOFs are different in STEM and HRTEM modes. In HRTEM mode, the thresholds are even lower at only dozens of electrons per square angstrom.^69^ To date, apart from some simulation studies,^70,71^ there have been no reports on atomic-resolution 4D-STEM ptychography of MOF materials.

In a recent study, Mary et al. acquired 4D-STEM data on a Hf-based metal–organic layer material with a total electron dose of 800 e^–^/Å^2^.^72^ Although this material is more stable than most MOFs, such a dose may have partially damaged its structure, which explains why only a 2.36 Å resolution was achieved in this study (Figure 2g). Another possible reason for not achieving atomic resolution is that the reconstruction algorithm (the SSB method) is not optimal.

Given the current status of 4D-STEM ptychography, the question arises: Is it possible to further reduce the electron dose to ultralow levels (e.g., <200 e^–^/Å^2^) to realize the potential advantages of 4D-STEM ptychography in imaging extremely sensitive materials? We believe this goal will likely be achieved if progress can be made in the following aspects.

Ultrafast electron detectors are required to reduce unnecessary exposure to the beam and lower the total electron dose. In our study on zeolites using a focused probe, the EMPAD detector with a 1000 fps frame rate could afford a total electron dose of 500–1000 e^–^/Å^2^, about 10 times higher than the critical dose that most MOFs can withstand. Thus, a reasonable estimate is that detectors with speeds above 10 000 fps are required for 4D-STEM of MOFs to avoid structural damage. Ultrafast detectors also minimize specimen drift that can adversely affect the subsequent reconstruction. Some prototype 4D-STEM detectors with speeds at this or even faster levels have been reported^28^ but are not yet widely available. The electron dose can also be reduced by using greatly defocused probes or a beam-blank technique.^73^

In addition to being very fast, the detector must have good sensitivity with a high detection quantum efficiency to ensure the high quality of the 4D-STEM data, the basis for successful ptychographic reconstruction. The detector performance at ultralow dose conditions is especially important when the material under study is extremely beam-sensitive. Although most 4D-STEM detectors allow the direct detection of electrons to eliminate readout noise, the data obtained at ultra-low-dose conditions certainly exhibit poor SNR. Whether atomic-resolution reconstruction can be successfully achieved using such noisy data remains unclear and requires careful, in-depth exploration.

The proper choice and optimization of the reconstruction algorithm are equally important as the advanced detector. The 4D-STEM ptychography reconstruction of highly beam-sensitive materials requires powerful algorithms that are robust to high-noise data and large defocus values. The ability of various existing algorithms to manage ultra-low-dose large-defocus data should be explored, and new algorithms that can meet these requirements should also be developed. Several methods for scanning coordinate correction^43,49^ and zone-axis correction^50^ have been developed to tolerate imperfect experimental conditions to a certain extent. These methods are potentially valuable for highly beam-sensitive materials that require rapid operation without time to perfect the imaging conditions.

Certain beam-sensitive materials exhibit enhanced beam tolerance at cryogenic temperatures. However, it remains challenging to achieve atomic-resolution ptychographic reconstruction using 4D-STEM data acquired at cryogenic temperatures, mainly because of the severe specimen drift caused by temperature instability, a common problem with the currently available cryo-TEM holders. The development of more stable cryo-TEM double-tilt holders is expected to facilitate high-resolution imaging of beam-sensitive materials using various (S)TEM modes including 4D-STEM ptychography.

In summary, atomic-resolution imaging of electron-beam-sensitive materials using 4D-STEM ptychography is a promising research area. For materials with moderate beam sensitivity, such as zeolites, 4D-STEM ptychography will become a routine method, offering advantages over conventional (S)TEM, including higher resolution, greater tolerance to specimen thickness and imperfectness of the focus, and additional resolving power in the direction of beam incidence. For materials with extremely high beam sensitivity, such as MOFs and hybrid perovskites, atomic-resolution 4D-STEM ptychography reconstruction is likely to be feasible despite some uncertainties. Achieving this challenging goal requires combining advanced detector technology with powerful reconstruction algorithms. If successful, imaging highly beam-sensitive materials will usher in a new era with remarkable improvements in precision and efficiency.

## Supporting Information Available

Transparent Peer Review report available (PDF)
