# Supplementary material for: 4D-STEM Ptychography for Electron-Beam-Sensitive Materials
Source: ACS Cent Sci. 2022 Nov 21;8(12):1579–88. doi: 10.1021/acscentsci.2c01137 (PMC9801507; doi:10.1021/acscentsci.2c01137)
Supplement: Supplementary file 1 — oc2c01137_si_001.pdf [file oc2c01137_si_001.pdf]

oc-2022-01137t.R1

Name: Peer Review Information for "4D-STEM Ptychography for Electron Beam–sensitive Materials"

First Round of Reviewer Comments

Reviewer: 1

Comments to the Author

Electron ptychography is a resurgent field of research with significant potential for super-resolution, multidimensional, and low-dose imaging. Its revival is due to the emergence of 4D-STEM enabled by advanced electron detectors. While the power of 4D-STEM ptychography has been demonstrated mainly by physicists, its applications in chemistry (i.e., imaging of chemical materials) have not been explored. In fact, although 4D-STEM ptychography is in principle an efficient low-dose imaging technique, especially suitable for imaging electron beam-sensitive materials, there is a lack of systematic studies on the limit of low electron dose that this technique can afford. Whether 4D-STEM ptychography can be used at electron doses as low as tens of electron per square angstrom, which is required by many chemical materials (e.g., MOFs, COFs, hybrid perovskites), remains an unanswered question.

In this context, the outlook article by Li et al. is a timely and significant contribution to the electron microscopy and chemistry communities. In this article, the authors introduce the fundamentals of 4D-STEM ptychography, elaborating its advantages over conventional imaging modes. They also summarize the current studies in this area and indicate the lack of studies of using 4D-STEM ptychography to image extremely beam-sensitive materials. Subsequently, they perform their own analysis, by simulation and experiments, to demonstrate the feasibility of applying this technique to materials with moderate sensitivity to the electron beam (e.g., zeolites). Lastly, they discuss the challenges of further extending this technique to extremely sensitive materials and the prerequisites needed for success.

Overall, this is a well-organized article and the analyses made are solid and sound. It provides constructive and insightful guidance for development of new imaging techniques for highly beam-sensitive materials. I recommend it to be accepted for publication in ACS Central Science, with the following minor changes.

1. There is no description on the meaning of “e-beam” in Fig. 1a, either in the main text or in the figure caption.

2. Add scale bar in Fig. 2c and scale bar descriptions for Figs. 2e and 2g in the figure caption.

3. In Fig. 3a, it is better to use 0.75 nm, 7.5 nm and 22.5 nm instead of 1 unit-cell, 10 unit-cells and 30 unit-cells.

4. Some references about the algorithms need to be added, for example, Opt. Express, 2020, 28, 28306 and Phys. Rev. B, 2014, 89, 064101.

Reviewer: 2

Comments to the Author

I have read this outlook article by Li et al with great interest. The article gave a nice introduction to the basic aspects of 4D STEM ptychography and several excellent examples in the studies of various materials. The authors also discuss the potential application of 4D STEM ptychography in imaging beam sensitive materials via simulation and experiments using zeolite as an example. The article is almost acceptable for publication in its current form. But I would still suggest a few minor changes for further improvement.

1. The authors mentioned a few interesting works combining ptychography with cryogenic temperature microscopy, but did not come back to this point in the outlook section. I would be interested in seeing a discussion on the possible new opportunities in imaging ultra-sensitive materials at atomic-resolution by combining cryo-EM with ptychography.

2. In the outlook section when discussing the possible solutions for further reducing the dose level, the authors seemed to suggest that the currently achievable low-dose level is limited by the speed of the defectors. Increasing the camera speed by a factor of 10 while maintaining the same probe current could help to reduce the dose level by a factor of 10. But isn't this the same if we simply reduce the probe current by a factor of 10 while using the same detector? Maybe the authors can help to clear out this confusion. In my opinion, with the state-of-the-art high-speed direct electron detectors, the unnecessary exposure to the beam may be caused and limited by the flyback time of the scan system instead of the camera speed, but I may be wrong.

3. As limited by the length of the article, the present paper may not go too much into the technical details, but it would be useful to refer the readers to some excellent recent review articles, e.g. Microscopy and Microanalysis (2019), 25, 563–582.

Author's Response to Peer Review Comments:

Dear Editor,

Thank you for your email on Oct 25, 2022 regarding our manuscript (oc-2022-01137t).

We are grateful to the two reviewers for their appreciation of our work and insightful comments. We provide our point-by-point responses to the reviewers' comments in the Appendix of this letter, and we have revised the manuscript accordingly. We believe that the clarifications and modifications we made in the revised manuscript fully address the reviewers' queries and concerns.

In addition, we have provided TOC image along with TOC SYNOPSIS at the end of the revised manuscript.

The pull quotes are uploaded in a separate word file.

We hope that you will find our revised manuscript compelling and suitable for publication in ACS Central Science.

Thank you again for your kind invitation.

Sincerely yours,

Yu Han

Point-by-point responses to the reviewers' comments

Reviewer #1:

Electron ptychography is a resurgent field of research with significant potential for super-resolution, multidimensional, and low-dose imaging. Its revival is due to the emergence of 4D-STEM enabled by advanced electron detectors. While the power of 4D-STEM ptychography has been demonstrated mainly by physicists, its applications in chemistry (i.e., imaging of chemical materials) have not been explored. In fact, although 4D-STEM ptychography is in principle an efficient low-dose imaging technique, especially suitable for imaging electron beam-sensitive materials, there is a lack of systematic studies on the limit of low electron dose that this technique can afford. Whether 4D-STEM ptychography can be used at electron doses as low as tens of electron per square angstrom, which is required by many chemical materials (e.g., MOFs, COFs, hybrid perovskites), remains an unanswered question.

In this context, the outlook article by Li et al. is a timely and significant contribution to the electron microscopy and chemistry communities. In this article, the authors introduce the fundamentals of 4D-STEM ptychography, elaborating its advantages over conventional imaging modes. They also summarize the current studies in this area and indicate the lack of studies of using 4D-STEM ptychography to image extremely beam-sensitive materials. Subsequently, they perform their own analysis, by simulation and experiments, to demonstrate the feasibility of applying this technique to materials with moderate sensitivity to the electron beam (e.g., zeolites). Lastly, they discuss the challenges of further extending this technique to extremely sensitive materials and the prerequisites needed for success.

Overall, this is a well-organized article and the analyses made are solid and sound. It provides constructive and insightful guidance for development of new imaging techniques for highly beam-sensitive materials. I recommend it to be accepted for publication in *ACS Central Science*, with the following minor changes.

Response: We are grateful to the reviewer for her/his appreciation of our work.

Comment #1: There is no description on the meaning of “e-beam” in Fig. 1a, either in the main text or in the figure caption.

Response: We thank the reviewer for pointing this out. We have replaced “e-beam” with “electron beam” in Fig. 1a.

Comment #2: Add scale bar in Fig. 2c and scale bar descriptions for Figs. 2e and 2g in the figure caption.

Response: We have modified Fig. 2 in the revised manuscript following these comments.

Comment #3: In Fig. 3a, it is better to use 0.75 nm, 7.5 nm and 22.5 nm instead of 1 unit-cell, 10 unit-cells and 30 unit-cells.

Response: We have made this change according to the reviewer's comment.

Comment #4: Some references about the algorithms need to be added, for example, Opt. Express, 2020, 28, 28306 and Phys. Rev. B, 2014, 89, 064101.

Response: We thank the reviewer for the suggested references, which have been cited in the revised manuscript.

Reviewer #2:

I have read this outlook article by Li et al with great interest. The article gave a nice introduction to the basic aspects of 4D STEM ptychography and several excellent examples in the studies of various materials. The authors also discuss the potential application of 4D STEM ptychography in imaging beam sensitive materials via simulation and experiments using zeolite as an example. The article is almost acceptable for publication in its current form. But I would still suggest a few minor changes for further improvement.

Response: We are thankful to the reviewer for her/his appreciation of our work.

Comment #1: The authors mentioned a few interesting works combining ptychography with cryogenic temperature microscopy, but did not come back to this point in the outlook section. I would be interested in seeing a discussion on the possible new opportunities in imaging ultra-sensitive materials at atomic-resolution by combining cryo-EM with ptychography.

Response: We thank the reviewer for this insightful comment. The combination of cryo-TEM with ptychography has been used for imaging biological specimens (e.g., Ref. 59). However, it remains challenging to achieve atomic resolution ptychographic reconstruction at cryogenic temperatures, mainly because the severe specimen drift at cryogenic temperatures adversely affects 4D-STEM data acquisition. Following the reviewer's comment, we briefly discuss this point in the outlook section of the revised manuscript.

Comment #2: In the outlook section when discussing the possible solutions for further reducing the dose level, the authors seemed to suggest that the currently achievable low-dose level is limited by the speed of the defectors. Increasing the camera speed by a factor of 10 while maintaining the same probe current could help to reduce the dose level by a factor of 10. But isn't this the same if we simply reduce the probe current by a factor of 10 while using the same detector? Maybe the authors can help to clear out this confusion. In my opinion, with the state-of-the-art high-speed direct electron detectors, the unnecessary exposure to the beam may be caused and limited by the flyback time of the scan system instead of the camera speed, but I may be wrong.

Response: We agree with the reviewer that increasing the camera speed by a factor of 10 has the same effect as reducing the probe current by a factor of 10. However, the probe current cannot be reduced unlimitedly because of the instrumental limitations. Taking the FEI Titan Cubed Themis Z, which we used for the experiments, as an example, the lowest probe current in STEM mode is 0.05 ~ 0.1 pA. Using such a low probe current, combined with a 0.4*0.4 $Å^2$ scanning step and 1 ms frame time, the total electron dose is 2000~4000 e/$Å^2$, which is still too high for extremely sensitive materials such as MOFs. In addition, when the probe current is at an extremely low level (i.e., < 0.1 pA), the aberrations becomes severe, which also adversely affect the data quality. Therefore, using a faster detector is a better option to further reduce the electron dose.

The reviewer is correct that flyback time has a remarkable influence on the electron dose level when the detector is extremely fast (e.g., when the dwell-time is less than 5 µs; Microsc. Microanal., 2022, 28, 1428-1436). However, such a high speed is not always necessary. As noted above, the combination of 0.1 pA probe current, 0.4*0.4 $Å^2$ scanning steps, and 10 µs frame time leads to an electron dose of 40 e/$Å^2$, which is low enough for most sensitive materials. Therefore, in the application scenarios discussed in this manuscript, the effect of flyback time is not crucial.

Comment #3: As limited by the length of the article, the present paper may not go too much into the technical details, but it would be useful to refer the readers to some excellent recent review articles, e.g. Microscopy and Microanalysis (2019), 25, 563–582.

Response: Following the reviewer's suggestion, we cite the recommended references (Ref. 24 and Ref. 25) and highlight them in the main text in the revised manuscript.
